# Multi-wavelength fluorescence imaging with a da Vinci Firefly—a technical look behind the scenes

**DOI:** 10.1007/s11701-020-01170-8

**Published:** 2020-11-11

**Authors:** Philippa Meershoek, Gijs H. KleinJan, Danny M. van Willigen, Kevin P. Bauwens, Silvia J. Spa, Florian van Beurden, Erik J. van Gennep, Alexandre M. Mottrie, Henk G. van der Poel, T. Buckle, Fijs W. B. van Leeuwen, Matthias N. van Oosterom

**Affiliations:** 1 Interventional Molecular Imaging Laboratory, Department of Radiology, Leiden University Medical Center, Leiden, The Netherlands; 2 Department of Urology, Netherlands Cancer Institute-Antoni van Leeuwenhoek Hospital, Amsterdam, The Netherlands; 3 Department of Urology, Leiden University Medical Center, Leiden, The Netherlands; 4 Orsi Academy, Melle, Belgium; 5 Department of Urology, Onze-Lieve-Vrouw Hospital, Aalst, Belgium

**Keywords:** Fluorescence-guided surgery, Multispectral fluorescence, Laparoscopic surgery, Robotic surgery, Multiplexing, Multi-wavelength fluorescence

## Abstract

The field of fluorescence-guided surgery builds on colored fluorescent tracers that have become available for different clinical applications. Combined use of complementary fluorescent emissions can allow visualization of different anatomical structures (e.g. tumor, lymphatics and nerves) in the same patient. With the aim to assess the requirements for multi-color fluorescence guidance under in vivo conditions, we thoroughly characterized two FDA-approved laparoscopic Firefly camera systems available on the da Vinci Si or da Vinci Xi surgical robot. In this process, we studied the cameras’ performance with respect to the photophysical properties of the FDA-approved dyes Fluorescein and ICG. Our findings indicate that multi-wavelength fluorescence imaging of Fluorescein and ICG is possible using clinical-grade fluorescence laparoscopes, but critical factors for success include the photophysical dye properties, imaging system performance and the amount of accumulated dye. When comparing the camera performance, the Xi system provided more effective excitation (adaptions in the light source) and higher detection sensitivity (chip-on-a-tip and/or enhanced image processing) for both Fluorescein and ICG. Both systems can readily be used for multi-wavelength fluorescence imaging of Fluorescein and ICG under clinically relevant conditions. With that, another step has been made towards the routine implementation of multi-wavelength image-guided surgery concepts.

## Introduction

Since its revival early this century, the field of fluorescence-guided surgery has rapidly progressed, in part due to the plurality of differently colored fluorescent tracers that have become available for clinical use [1]. The near-infrared (NIR) dye indocyanine green (ICG; λ_ex max_ = 800 nm, λ_em max_ = 820 nm) is routinely applied as dye for fluorescence-guided surgery and has been the dye to serve as focal point in the development of most commercial fluorescence-guided surgery cameras [2]. There are also early clinical examples that make use of the NIR dyes IRDye 800CW (λ_ex max_ = 783 nm, λ_em max_ = 801 nm) and ZW800-1 (λ_ex max_ = 770 nm, λ_em max_ = 788 nm) [3, 4]. Interestingly, next to NIR dyes, visible dyes, such as Fluorescein (λ_ex max_ = 488 nm, λ_em max_ = 515 nm), have been successfully employed in various surgical guidance settings: FITC, CEA-FITC and Folate-FITC [5–10]. Clinical examples that use (far-)red fluorescence to guide surgical excisions, using, for example, methylene blue (λ_ex max_ = 670 nm, λ_em max_ = 680 nm), Protoporphyrin IX (λ_ex max_ = 405 nm, λ_em max_ = 635 nm) and GE-137 (λ_ex max_ = 645 nm, λ_em max_ = 661 nm) [9–13], complete the list. Some of these applications contradict the popular (theoretical) notion that suggests that only NIR dyes are of value for surgical guidance due to spectral advantages, such as invisibility to the human eye and tissue penetration up to 1 cm [14]. Hence, the successful in vivo implementation of non-NIR dyes underscores that in practice, not only the emission wavelengths impact on the usability, but a far more complex combination of light source/camera settings and photophysical dye properties actually dictate a dye’s clinical potential.

The realization that different fluorescent emissions could provide value during fluorescence-guided surgery applications provides the possibility of exploring multi-wavelength (so-called multispectral, multiplexing or multi-color) surgical guidance applications [15], where different anatomical structures are visualized during surgery (e.g. tumor, lymphatics and nerves) [16–22]. While routine in microscopy, fluorescence multiplexing has, to date, only found limited use in vivo. Intriguingly, a previous study wherein Fluorescein and ICG were both implemented in a robot-assisted laparoscopic surgery setting [9, 23] revealed that both dyes could be visualized simultaneously in vivo. However, as the dyes could be visualized with seemingly similar intensities, the requirements for dye visibility under in vivo conditions remained unclear.

**Fig. 1:**
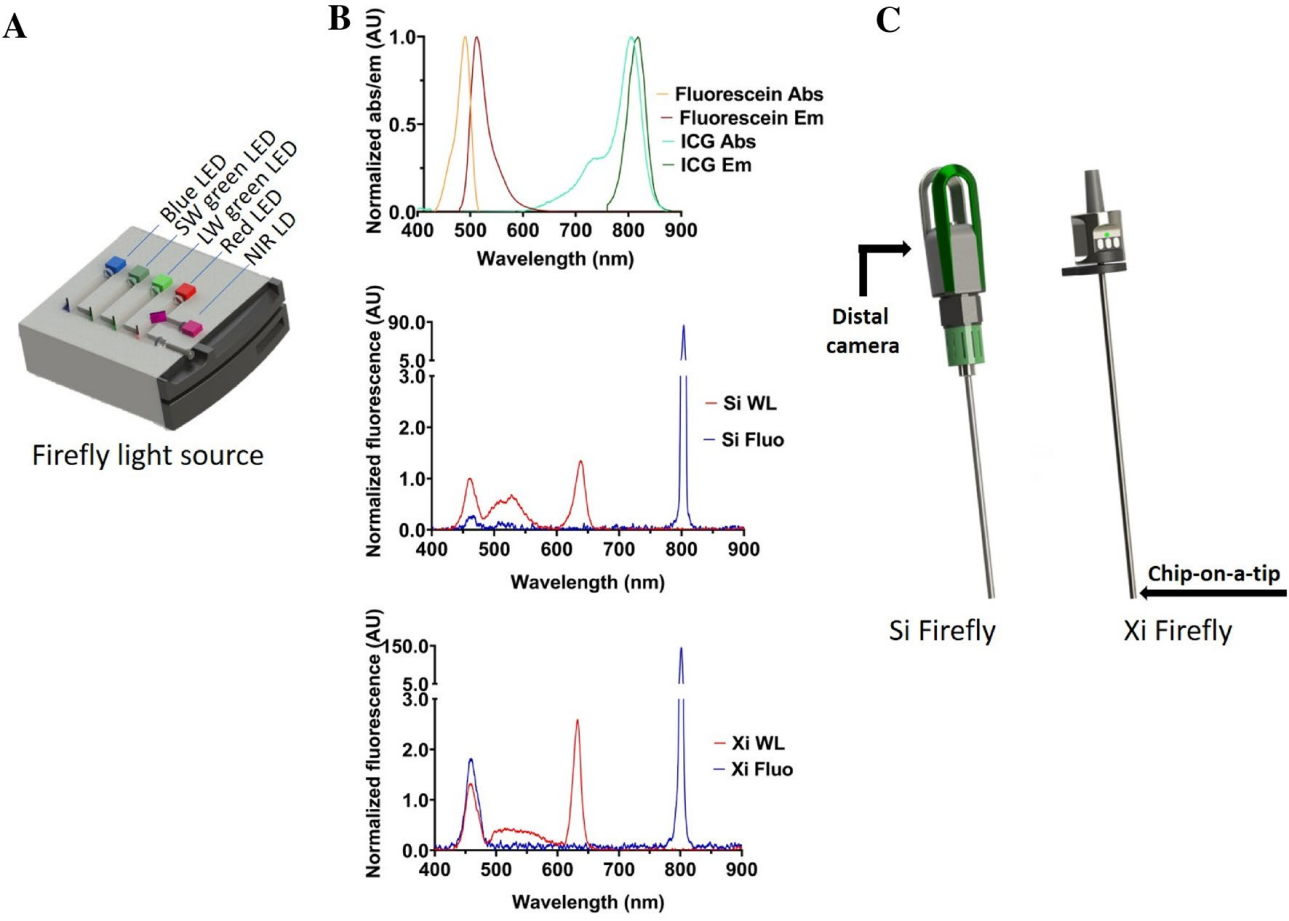
Hardware components of the da Vinci Si and Xi vision cart systems. **a** schematic representation of the light source, **b** top: excitation and emission spectra of Fluorescein and ICG, middle: Spectral properties of the Si light source in white light mode (red) and fluorescence mode (blue), bottom: Spectral properties of the Si and Xi light sources in white light mode (red) and fluorescence mode (blue), **c** schematic representation of the Si and XI Firefly laparoscopes and their detector location. *LED* light-emitting diode, *SW* short-wave, *LW* long-wave, *NIR* near-infrared, *LD* laser diode, *Abs* absorbance, *Em* emission, *WL* white light mode, *Fluo* fluorescence mode

With this study, we aimed to address the direct relation between the light source/camera settings of the Firefly laparoscopic imaging systems of the da Vinci Si and Xi surgical robots and their performance with respect to the photophysical properties of the FDA-approved dyes Fluorescein and ICG (Fig. 1). In doing so, we also directly compare a conventional laparoscopic camera arrangement (the Si Firefly system, where the camera is located outside of the patient at the end of a scope) to an endoscopy-derived chip-on-a-tip arrangement (Xi Firefly system, where the camera is located inside of the patient).

## Methods

### Fluorescence camera systems

The Firefly camera systems used for this study were integrated into robotic surgery systems (da Vinci Si and Xi; Intuitive Surgical Inc.). Both vision carts use an externally placed light source containing an arrangement of 4 LEDs to create a well-balanced white light illumination during normal surgery (i.e. white light imaging mode) and a laser source for excitation of ICG during fluorescence imaging (Fig. 1a, b) [24]. The camera system, however, differs substantially between the two systems. The Si system uses an externally placed camera, which images inside the patient via a laparoscope optic, while the Xi system relies on a chip-on-a-tip configuration that is directly placed inside the patient (Fig. 1c). For the Si system, images could be recorded in three modes: white light, unprocessed fluorescence and processed fluorescence imaging. For the Xi system, images could only be recorded in white light and processed fluorescence imaging modes.

### Fluorophore photon flux

To determine the theoretical differences in fluorophore brightness, the photon flux of both Fluorescein (Fluorescite, 100 mg/ml solution for injection, S.A. Alcon-Couvreur NV, Belgium) and ICG (ICG-Pulsion, 25 mg vial; Pulsion Medical Systems, Munich, Germany) were estimated based on their photophysical properties (i.e. molar extinction coefficient, fluorescence lifetime [25] and quantum yield). Since these properties can be influenced significantly by the fluorophore-dissolving medium, a human serum albumin (HSA) medium was chosen to approximate the in vivo application.

#### Molar extinction coefficient of Fluorescein and ICG

A 4 mM stock solution of fluorophore (i.e. either Fluorescein or ICG) was prepared in a DMSO-d6 (800 µL) medium with 4 mM ethylene carbonate. The exact concentration was verified by NMR using ethylene carbonate as internal standard [26]. This was further diluted with H_2_O containing 100 mg/mL HSA to obtain a final concentration series of 5, 2.5, 1.2, 0.6, and 0.3 µM. Absorption spectra were measured 10 min after preparation using 1 mL disposable plastic cuvettes (*l* = 1 cm; Brand, Germany) and an Ultrospec 3000 UV/Vis spectrometer (GE Healthcare Chicago, Illinois, USA). The maximum absorbance was determined, where after the molar extinction coefficient ε was determined from the linear regression coefficient.

#### Quantum yield of Fluorescein and ICG

Fluorescein and ICG were each dissolved in H_2_O containing 100 mg/mL HSA to obtain 3 mL 0.5 µM in 4.5 mL cuvettes (*l* = 1 cm; Kartell, Germany). Fluorescence intensities were measured using a Perkin Elmer LS-55 fluorescence spectrometer (Waltham, Massachusetts, USA) using excitations at λ_ex_ = 460 nm for Fluorescein and λ_ex_ = 736 nm for ICG and collecting the signal from 350 to 750 nm and 600 to 900 nm, respectively. To determine the quantum yield Q_F_, the absorbance at the excitation wavelengths was correlated with the integrated fluorescent emission. The regression coefficient of the resulting plots was compared to the regression coefficients of dye solutions with known quantum yields: Fluorescein in 0.1 M NaOH (Q_F_ = 93%) [27] or ICG in DMSO (Q_F_ = 12%) [28].

#### Relative photon flux of Fluorescein and ICG

The relative photon flux of Fluorescein and ICG was calculated as shown by Chin et al. [29]. For one, dyes absorb excitation light based on their optical cross section (σ; cm^2^/molecule). This feature was calculated from the extinction coefficient, (ε; L·mol^−1^·cm^−1^) using the following formula, where *N*_*a*_ is Avogadro’s constant (*N*_*a*_ = 6.022·10^23^ molecules/mol):

1 $$\sigma = \varepsilon \cdot 1000\left( {\frac{{cm^{3} }}{L}} \right) \cdot \frac{ln10}{{N_{a} }}$$

By multiplying σ with the excitation intensity (*I*_*0*_*)*, the rate constant of absorption (*k*_*a*_) for a single fluorophore molecule can be calculated. Here, *I*_*0*_ is given as:

2 $$I_{0} = P \cdot \left( {\frac{h \cdot c}{\lambda }} \right)^{ - 1}$$

with *P* the excitation power (W/cm^2^), *h* Planck’s constant (*h* = 6.626·10^–34^ J·s), *c* the speed of light in vacuum (*c* = 2.998·10^10^ cm/s) and *λ* the excitation wavelength (in cm). For the relative comparison, an equal excitation power *P* was assumed of 1·10^–3^ W/cm^2^. *λ* was chosen as 488 nm for Fluorescein and 800 nm for ICG. To subsequently determine the fraction of molecules in the excited state (*w*), the rate constant (*k*_*f*_) for relaxation was also included, which is the inverse of the fluorescence lifetime (τ_*1/2*_ in s). *w* was calculated when these features are combined in the formula:

3 $$w = \frac{{k_{a} }}{{\left( {k_{a} + k_{f} } \right)}}$$

By further combining the outcome of this formula with the fluorescence quantum yield Q_F_, the conversion of excitation light into an expected average photon flux per fluorophore molecule (in photons/s) could be calculated:

4 $$I_{E} = Q_{f} \cdot k_{f} \cdot w$$

In this calculation, the influence of tissue attenuation and autofluorescence were neglected and the presence of an excess of albumin preventing fluorophore self-quenching was assumed.

### Fluorophore luminescence quantification

The relation between the fluorophore concentration and the luminescence signal was evaluated using a dilution series of Fluorescein and ICG. Both dilution series were prepared in black well plates (96-wells, Cellstar; Greiner Bio-One GmbH, Frickenhausen, Germany) with each well containing 50 μL of solution. Fluorescein was converted into a 5.0 mg/mL (13.3 × 10^−3^ M) starting solution. This solution was diluted 1:1 with a 100 mg/mL HSA in water in 35 steps down to 1.46 × 10^−10^ mg/mL (3.87 × 10^−13^ M). ICG was diluted in the same fashion starting from a 5.0 mg/mL (6.45 × 10^−3^ M) solution down to 1.46 × 10^−10^ mg/mL (1.88 × 10^−13^ M). Subsequently, the fluorescence intensities of both dye dilution series were measured using an IVIS preclinical camera system (IVIS Spectrum, Xenogen Corporation, San Francisco, CA, USA). For Fluorescein, λ_ex_ = 465 nm and λ_em_ = 540 nm were used, for ICG, λ_ex_ = 745 and λ_em_ = 800 nm. Quantification of these measurements was performed using the accompanying IVIS Living Image 3D analysis software (version 1.0; Xenogen Corporation). With this software, the average radiance (in photons/s/cm^2^/sr) was determined for all dye containing wells. These measurements were background corrected using a well with only HSA solution present and normalized per fluorophore.

### Spectral characterization of the Firefly illumination sources

**Fig. 2:**
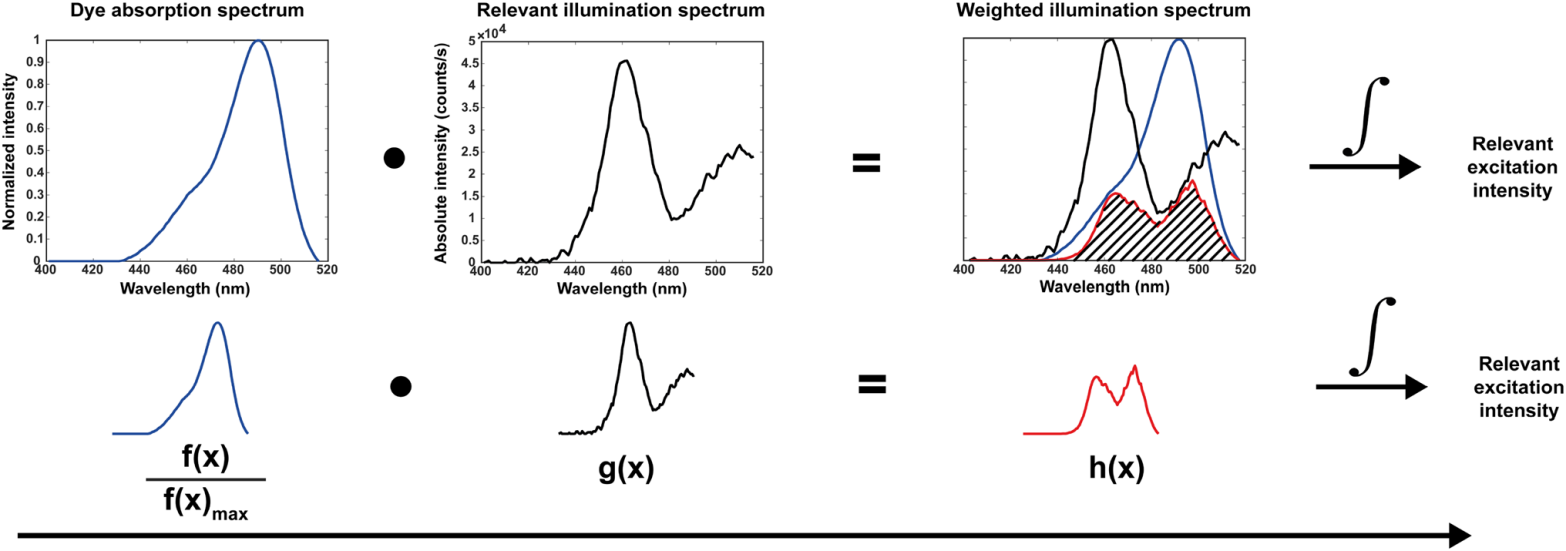
Illustration of the relevant excitation intensity calculations (formula 5), displaying the spectral overlap of the excitation light source and the fluorescent dye absorbance. Example with Fluorescein: the normalized dye absorption spectrum (blue) multiplied by the illumination spectrum (black) results in a weighted illumination spectrum (red). The integral of this weighted illumination spectrum (red with dashed area under the curve) provides the relevant excitation intensity

To study the efficiency with which the Firefly illumination source, incorporated in either the Xi or Si system, can excite both Fluorescein and ICG, the illumination characteristics were studied using a Horiba Jobin Yvon VS140 linear array fiber spectrometer (Horiba Ltd., Kyoto, Japan) with a 1 m optical fiber (M15L01, Thorlabs Inc., Newton, NJ, USA). This was done for the Si and Xi systems in both white light and fluorescence imaging modes. Subsequently, the effective overlap between the dye absorption spectrum (*f*(*x*), in counts/s) and the Firefly excitation light spectrum (*g*(*x*), in counts/s) at the specific wavelengths (*x*, in nm) was determined. Estimation of the effective excitation intensity (${Ex}_{eff}$ in counts × nm/s), was realized using the following formula:

5 $$Ex_{eff} = \int {\frac{f\left( x \right)}{{f\left( x \right)_{max} }} \cdot g\left( x \right) \cdot dx} = \int {h\left( x \right) \cdot dx}$$

where $\frac{f(x)}{{f(x)}_{max}}$ presents the normalized dye absorption spectrum and *h*(*x*) is the weighted excitation light spectrum. These calculations were performed in MATLAB (MathWorks Inc., Natick, MA, USA). An illustration of this calculation is shown in Fig. 2.

By combing the effective excitation factors (*Ex*_*eff*_) of the Firefly systems with the dye photon fluxes, an estimation for the fluorophore-expected-light-yield was determined using the following formula:

6 $${\text{Fluorophore - expected - light - yeild = photon flux}}\cdot{\text{ Ex}}_{{{\text{eff}}}}$$

### Firefly detection sensitivity for Fluorescein and ICG

The detection sensitivity of both the Si and Xi Firefly systems was determined using Fluorescein and ICG dilution series similar to those used for luminescence quantification. Evaluation was performed in a setup wherein the two different Firefly cameras were fixed perpendicularly at a 1 cm distance from the well-plate surface. During imaging, the ambient light was dimmed, and images were recorded at the different camera settings (i.e. white light, unprocessed fluorescence and processed fluorescence imaging with the Firefly Si; white light and processed fluorescence with the Firefly Xi).

### In vivo evaluation

In vivo performance of multispectral fluorescence imaging using Fluorescein and ICG was evaluated in 5 male pigs, as previously described [23]. To allow independent visualization of the lymphatics draining the legs and the prostate, Fluorescein was injected into the lower limb, while ICG-nanocolloid was injected directly into the prostate. For Fluorescein, a 5 mL 100 mg/mL solution was used, injected with either 2 deposits (subcutaneous and intramuscular) in a single leg or with 4 deposits (subcutaneous and intramuscular) in both legs of the animal. ICG-nanocolloid was injected as a 2 mL 0.125 mg/mL solution using 2–4 deposits.

## Results

### Fluorophore photon flux

From the differences in estimated photon flux, it becomes evident that in a surgically relevant environment (i.e. HSA containing medium), the number of emitted photons per second by ICG is about twice as high as by Fluorescein (Table 1). It is important to note that under these HSA conditions, the quantum yield for Fluorescein (12%) is significantly lower than what is often reported (93% [27]).

**Table 1:** Photophysical properties Fluorescein and ICG

Fluorescent dye	Extinction coefficient ε (L·mol^−1^·cm^−1^)	Fluorescence lifetime *τ*_*1/2*_ (ns)	Quantum yield Q_F_ (%)	Estimated photon flux (photons/s·molecule)
Fluorescein	0.4 × 10^5^ (HSA)	4 (H_2_O, [25])	12 (HSA)	4.5 × 10^–2^
ICG	1.8 × 10^5^ (HSA)	0.97 (DMSO, [25])	5 (HSA)	13.97 × 10^–2^

### Fluorophore luminescence quantification

**Fig. 3:**
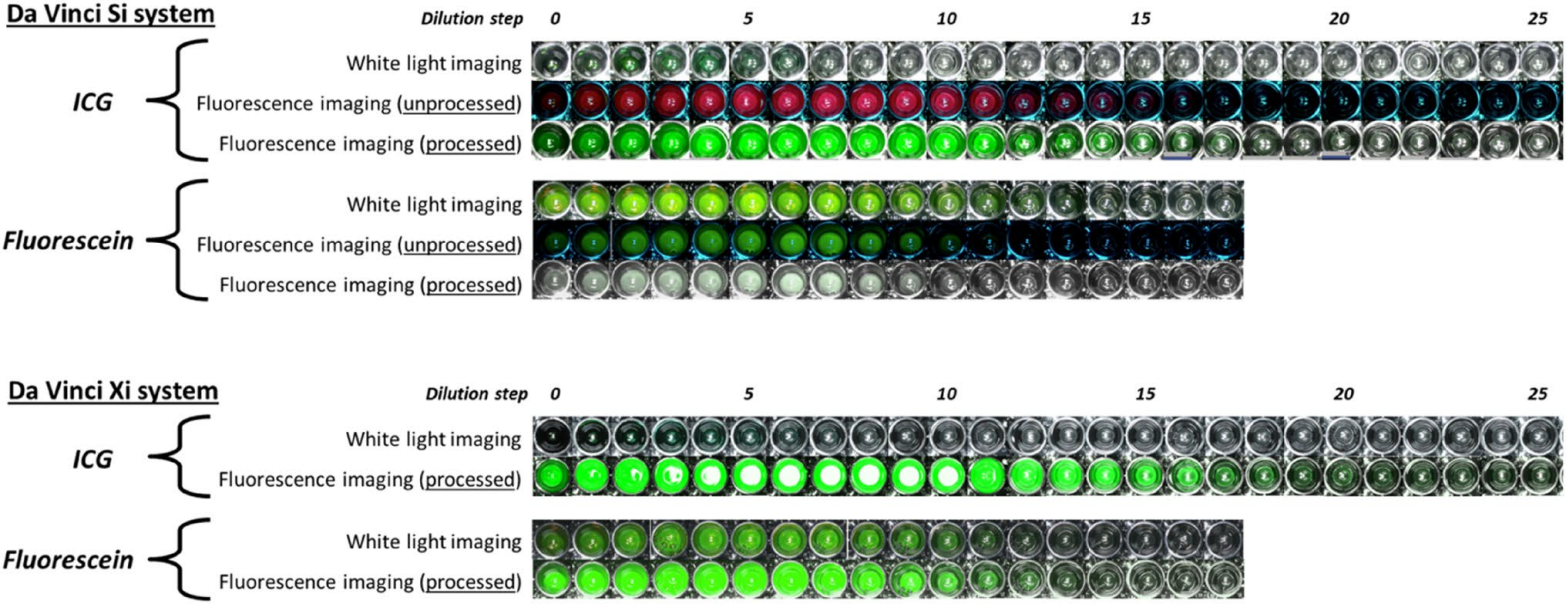
Detection sensitivity. Dilution series of Fluorescein and ICG imaged using the Si Firefly system (top) and the Xi Firefly system (bottom) showing white light and fluorescence imaging

**Fig. 4:**
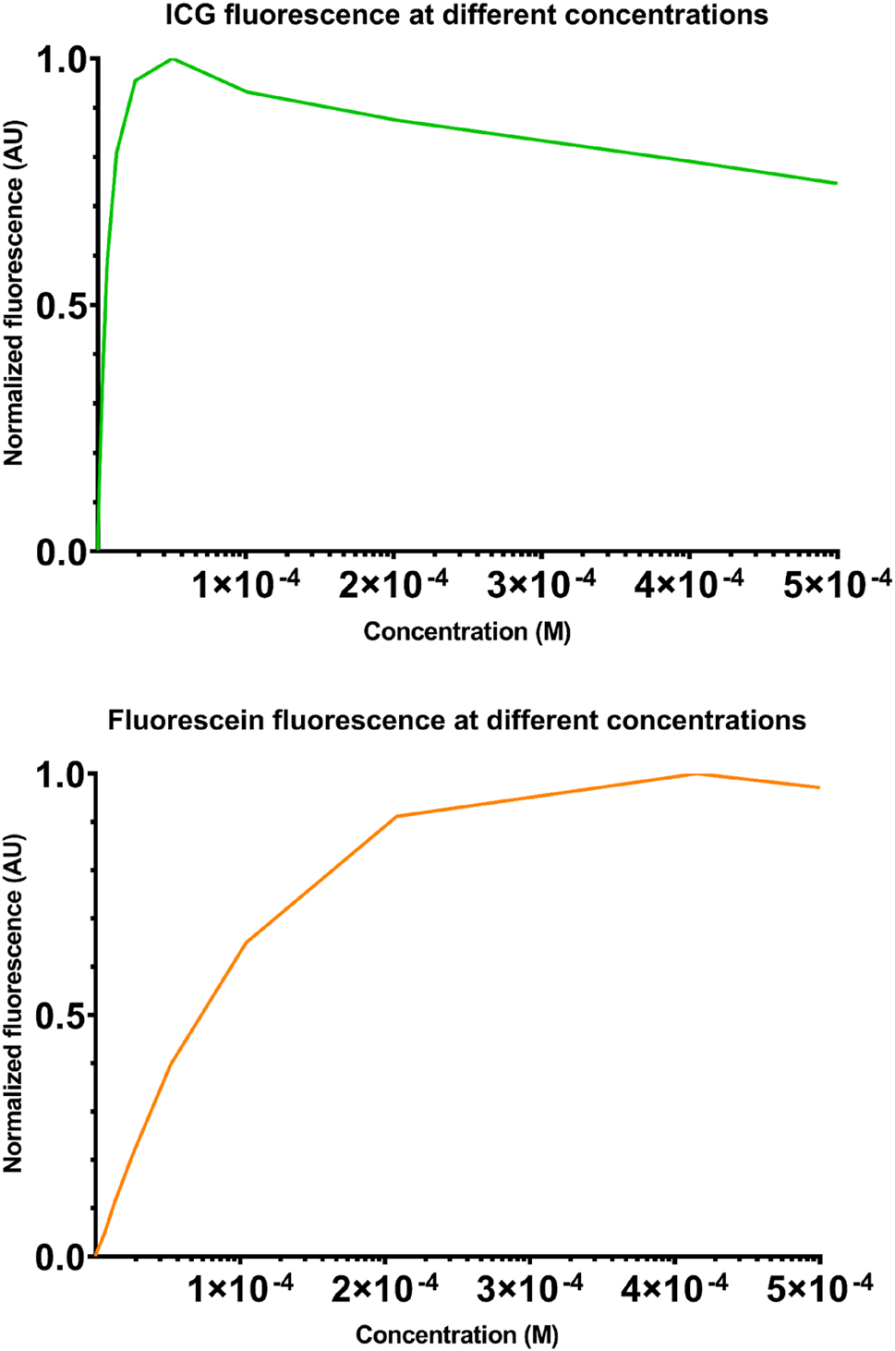
Concentration dependent fluorescence intensities for Fluorescein and ICG. For ICG self-quenching starts to occur around 5 × 10^–5^ M. For Fluorescein, self-quenching only starts around much higher values: 4 × 10^–4^ M

Quantification of the fluorescence intensities of the Fluorescein-HSA and ICG-HSA well-plate dilution series revealed a clear concentration dependence for both the dyes (Figs. 3 and 4). At low concentrations, detected luminescence rapidly increased with concentration. However, at higher concentrations, the photon flux flattened and eventually even started degrading again. This is most likely a result of self-quenching and was more prominent for ICG (turning point around 5 × 10^–5^ M) as compared to Fluorescein (turning point around 4 × 10^–4^ M). This result indicates Fluorescein can be used more efficiently at high concentrations.

### Spectral characterization of the Firefly Si vs. Xi illumination sources

Figure 1b shows the spectral properties of the Firefly Si and Xi light sources with respect to the Fluorescein and ICG absorption and emission curves. Both Si and Xi systems displayed a very similar spectrum formed by a combination of different LEDs, for imaging of the surgical context, and a NIR laser for ICG excitation. However, illumination levels of the individual components did differ. Especially the blue illumination and NIR illumination of the Xi system in fluorescence imaging mode were higher as compared to the Si system in fluorescence imaging mode (Fig. 1b). In the white light modus of both systems, there is a clear overlap between the blue illumination peak (λ_ex max_ =  ~ 460 nm) and the absorption spectrum of Fluorescein (λ_ex max_ = 488 nm; Fig. 1b). This explains why Fluorescein is excited in the white light imaging setting of both Si and Xi systems. In the fluorescence mode of both systems, a small (relative to NIR illumination) but distinct blue emission peak remains visible, still supporting the excitation of Fluorescein. However, as shown by the calculated effective excitation intensities of the Si system, the blue-light intensity in fluorescence mode was five times lower compared to its intensity in the white light mode (Table 2). For the Xi system, the effective Fluorescein excitation was very similar in fluorescence and white light imaging modes (Table 2). In the fluorescence modus, the NIR illumination peak (λ_ex max_ =  ~ 800 nm) overlaps with the absorption spectrum of ICG (λ_ex max_ = 800 nm). In the Xi system, the ICG excitation source appeared to be roughly two times more intense than in the Si system, while maintaining its general characteristics (Table 2). When compared to ICG excitation in the fluorescence imaging mode, the effective Fluorescein excitation was 117 times lower for the Si system and 40 times lower for the Xi system. When taking the twofold higher photon flux for ICG (Table 1) into account, the normalized fluorophore-expected-light-yield with these systems is: Fluorescein^Si^ = 1, ICG^Si^ = 304.4, Fluorescein^Xi^ = 5 and ICG^Xi^ = 541.2 (values were normalized to Fluorescein application with the Si system). The relative Fluorescein light yield should, therefore, be five times higher for the Xi system as compared to the Si system. For both systems, the laser-excited ICG light yield was shown to exceed that of LED-excited Fluorescein: ~ 300-times with the Si and ~ 500-times with the Xi.

**Table 2:** Relative excitation intensity of Fluorescein and ICG for Firefly Si and Firefly Xi

Setting	Relevant excitation intensity for Fluorescein (counts × nm/s)	Relevant excitation intensity for ICG (counts × nm/s)
Si white light	6.76 × 10^5^	8.08 × 10^4^
Si fluorescence	1.32 × 10^5^	1.54 × 10^7^
Xi white light	6.81 × 10^5^	9.04 × 10^4^
Xi fluorescence	6.89 × 10^5^	2.75 × 10^7^

### Firefly Si vs. Xi detection sensitivity for Fluorescein and ICG

Evaluation of Fluorescein and ICG dilution series with the Si and Xi Firefly using the different white light and fluorescence imaging settings (Fig. 3) allowed us to determine the lowest detectable fluorophore concentrations (Table 3), not only revealing differences between unprocessed and processed fluorescence intensities for the Si Firefly (respectively, ^Si^_unprocessed_ and ^Si^_processed_) but also between ^Si^_processed_ and the processed intensities found for the Xi Firefly (^Xi^_processed_). When combined with the fluorescence luminescence quantification, a similar detection sensitivity was found for Fluorescein^Si^_unprocessed_ and Fluorescein^Si^_processed_: a fluorophore concentration of 1.04 × 10^–4^ M, providing a normalized radiance of 65.0 × 10^–2^. The Fluorescein^Xi^_processed_ sensitivity was 1.30 × 10^–5^ M (normalized radiance: 11.1 × 10^–2^). This converts to a rough relative light-level factor that is 6-times weaker compared to Fluorescein^Si^_processed_. When related to the differences in fluorophore-expected-light-yield (i.e. 5 times higher for Xi compared to Si Fluorescein imaging; Table 2), this indicates the Xi system benefits from an additional gain in Fluorescein sensitivity due to image processing and/or detector sensitivity.

**Table 3:** Camera sensitivities and minimum detected radiance in fluorescence imaging mode

	Fluorescein		ICG	
	Minimum detected concentration (M)	Normalized minimum detected radiance (a.u.)	Minimum detected concentration (M)	Normalized minimum detected radiance (a.u.)
Si_unprocessed_	1.04 × 10^–4^	65.0 × 10^–2^	1.58 × 10^–6^	20.0 × 10^–2^
Si_processed_	1.04 × 10^–4^	65.0 × 10^–2^	1.97 × 10^–7^	4.70 × 10^–2^
Xi_processed_	1.30 × 10^–5^	11.1 × 10^–2^	2.46 × 10^–8^	1.15 × 10^–2^

The ICG^Si^_processed_ detection of 1.97 × 10^–7^ M (normalized radiance: 4.70 × 10^–2^) was about four times more sensitive than the ICG^Si^_unprocessed_ detection (1.58 × 10^–6^ M; IVIS light level: 20.0 × 10^–2^), suggesting that the processing yields a fourfold artificial enhancement of the detection sensitivity. With a similar calculation, the relative fluorescence imaging sensitivity of ICG^Xi^_processed_ (2.46 × 10^–8^ M; IVIS light level: 1.15 × 10^–2^) was found to be fourfold higher than for ICG^Si^_processed_. When the differences in the fluorophore-expected-light-yield (i.e. roughly 2 times higher for Xi compared to Si; Table 2) are considered, the additional yield in ICG sensitivity for the Xi system suggests a difference in processing and/or detector sensitivity between the two vision carts. Given the difference in the location of the detector between the Xi and Si system, the sensitivity difference could possibly also be contributed to the location of the detector (Si: camera at the end of the laparoscope vs. Xi: chip-on-a tip).

### In vivo multi-wavelength imaging

**Fig. 5:**
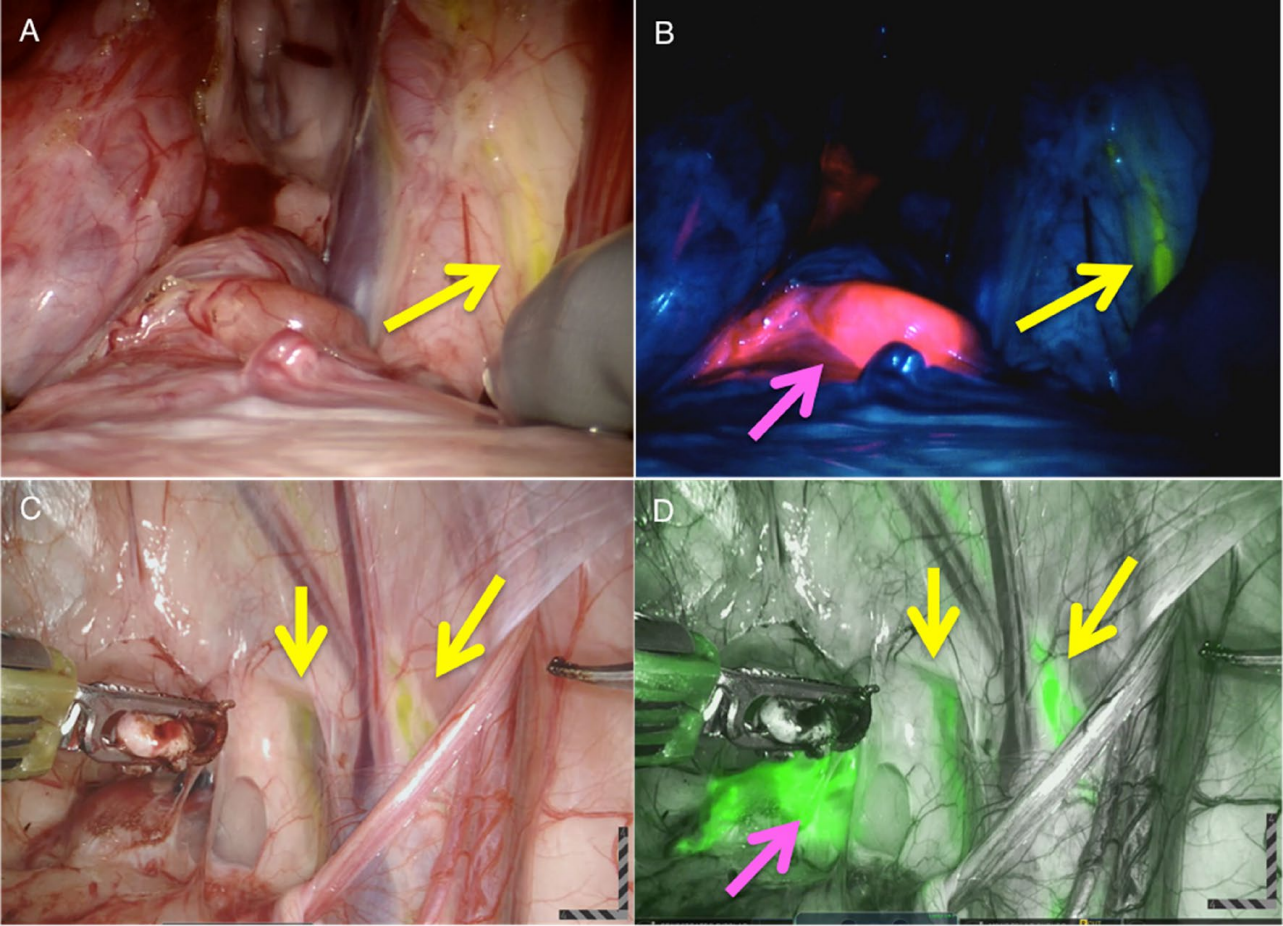
Multispectral imaging of Fluorescein and ICG in vivo with the Firefly Si and Xi. **a** White light (WL) image obtained with the Si Firefly showing a lymphatic vessel (yellow arrow) containing Fluorescein (in yellow) and **b** the same in vivo features visualized with fluorescence settings (Si_unprocessed_) revealing a lymph node (pink arrow) containing ICG (in pink) as well. **c** WL image obtained with the Xi Firefly showing two lymphatic vessels containing Fluorescein and **d** the same in vivo features visualized with fluorescence settings (Xi_processed_) showing both fluorescence signatures in green

In all 5 pigs, both ICG (in the ICG-nanocolloid formulation) and Fluorescein could be visualized in one and the same image (Fig. 5), a further confirmation that both Fluorescein and ICG can be visualized in vivo using the Firefly fluorescence imaging systems. Lymphangiography with a relatively high concentration of Fluorescein supported identification of lymphatics draining the legs, while specific nodal accumulation of ICG-nanocolloid in relatively low concentrations helped visualize the sentinel lymph nodes of the prostate [23]. The white light imaging mode of both the Si and Xi system already supported visual detection of Fluorescein. In the processed fluorescence images of the Xi system, both the Fluorescein and ICG signal were displayed as bright green, which complicated the differentiation between the two lymphatic drainage patterns in this mode (Fig. 5). However, the emissions could be clearly separated based on the unprocessed fluorescence images of the Si system (Fluorescein in yellow/green and ICG in pink/red; Fig. 5). As evident from Tables 1, 2 and 3, both Si and Xi Firefly cameras apply much higher excitation power for ICG as compared to Fluorescein, while ICG photon flux also exceeds that of Fluorescein in HSA medium. Nonetheless, similar visibility for both dyes was achieved in vivo using either system (Fig. 5). From this, it can be deduced that the dye concentrations in the depicted lymphatic structures must have differed. As was previously derived from clinical experience using ICG-nanocolloid, a typical median ICG dose value found in the sentinel nodes of prostate cancer patients is 2.86 × 10^–6^ M [30]. Since quantitative evaluation revealed that the minimal detectable Fluorescein concentration with the Si system in fluorescence mode was about 1.04 × 10^–4^ M (Table 3), it can be concluded that a Fluorescein dose of at least 1.04 × 10^–4^ M must have been present in the lymph nodes at the time of imaging. This converts to a rough concentration difference of  ≥ 10^2^. With the increased Fluorescein excitation of the Xi system (Tables 2 and 3), this difference was reduced to ≥ 10.

## Discussion

In this study, the technical background of in vivo multi-wavelength fluorescence imaging using  Fluorescein and ICG and clinically available fluorescence cameras of the da Vinci Si and Xi robotic platforms was evaluated. Our findings indicate that multiplexing of fluorescent tracers using clinical modalities, such as the Firefly-camera is possible, but that detectability is an intricate interplay of photophysical dye properties, vision cart settings, optics, and the concentration of dye with relation to the in vivo visibility. Although the Si and Xi Firefly systems are specifically designed for imaging of ICG, our findings underline that their imaging properties also facilitate in vivo Fluorescein imaging.

Our findings clearly reveal a difference in performance between the two camera systems and for the two dyes. With regard to the photophysical properties of the FDA-approved dyes ICG and Fluorescein we found: (1) The ICG photon flux in a surgically relevant environment (i.e. HSA medium) is roughly 2 times higher compared to Fluorescein and (2) Fluorescein can be used more efficiently in higher concentrations since self-quenching starts at roughly 10 times higher concentrations with respect to ICG. Differences were also found with regard to the fluorescence imaging settings of the Si and Xi vision carts. Starting with the light source properties: (1) The effective excitation of ICG is much higher than of Fluorescein for both systems (~ 117 times and ~ 40 times with the Si and Xi light source, respectively); (2) In fluorescence imaging mode, the Xi light source has 5 times more effective Fluorescein excitation as compared to the Si system; (3) The NIR laser in the Xi light source provides a 2 times more effective ICG excitation as compared to the Si light source. A similar trend was observed for the camera systems: (1) In fluorescence imaging mode, the Xi camera system is roughly 6 times more sensitive for Fluorescein detection as compared to the Si system; (2) The Xi camera is about 4 times more sensitive in ICG imaging as compared to the Si camera. Overall, this means the technical data indicate the Xi vison cart can be considered the superior of the two. Nevertheless, both systems effectively support in vivo imaging of Fluorescein. By comparing the Si and Xi Firefly systems, we also made a direct comparison between a conventional laparoscopic camera configuration (camera at the end of a scope; Si) and an endoscopy-derived chip-on-a-tip configuration (Xi). It seems that at least part of the improved performance characteristics of the Xi system can be contributed to the intensity of the light source. Nevertheless, the discrepancy between the twofold increase in ICG excitation and the fourfold increase in detection sensitivity, suggests that reducing the light path between the fluorescent source and detector could have a positive impact on the camera performance. It is not clear how much and/or which part of this intensity improvement is related to the image processing software that the system uses or an improvement in the detector sensitivity.

In the area of multi-wavelength fluorescence-guided surgery, there is much debate about the requirement of realizing fully integrated multi-color fluorescence images (as shown in this study) versus sequential image acquisition as was shown previously using an alternative system [9, 31]. During the surgical implementation of Firefly fluorescence guidance, it is routine that the white-light mode is used during most of the procedure, since white light allows for the best visual inspection of the surgical field, while the fluorescence mode is only used when better discrimination of the target tissue is needed, often following surgical exposure of the tissue. In a similar fashion, sequential imaging of Fluorescein and ICG has helped to clinically differentiate between (target) tissues [9, 17, 32]. However, simultaneously depicting two fluorescence tracers over an anatomical background (Fig. 5), eases the visual separation of these different (target) tissues within the surgical field. This suggests that both options could have potential. What is of great importance, however, is that color-coding-based differentiation of the fluorescence signals (as was observed in the unprocessed Si images) improves interpretation compared to a situation where both fluorescent emissions are depicted in the same color (Fig. 5).

During this study, clinically approved dyes were used in combination with FDA-approved camera systems, whereby the latter were designed specifically for ICG imaging. With regard to the i*n vivo* utility for multi-wavelength surgical guidance, two utility-related observations could be made: (1) Despite being optimized for ICG, both Firefly systems are able to simultaneously visualize Fluorescein and ICG in vivo; (2) To obtain similar visualization of Fluorescein and ICG-nanocolloid in vivo, using the current Si and Xi systems, a rough concentration increase of at least 10^2^-times (Si) or 10 times (Xi) was necessary for Fluorescein. With a clinical Fluorescein dose of 6.7 mg/kg (or 20 nmol/kg) [33] and dose of 0.3 mg/kg (or 0.4 nmol/kg) [34] for ICG, this feature is not limiting. This means that the current multispectral imaging setup can be readily translated into human subjects. Additionally, as showed in a different study, a slightly modified Si Firefly system also allowed for the imaging of the far-red dye Cy5 [35]. Combined these points indicate that the implementation of future technical refinements to the Firefly systems could result in a complete multispectral camera system that is fully compatible with the da Vinci surgical robot. This feature, combined with the wide install base of Intuitive, could help disseminate the use of multi-wavelength image guidance.

## Conclusion

The ability to clearly image both Fluorescein and ICG in a surgical setting using da Vinci clinical-grade vision carts (Firefly Si (camera at the end of a laparoscope) and Xi (chip-on-a-tip arrangement)) underscores that wavelength-dependent tissue penetration does not fully dictate in vivo utility, but a more complex balance including the dye photophysical properties, dye concentration/accumulation and fluorescence imaging settings. By underscoring that visible dyes have a value during in vivo imaging applications as well, another step has been made towards the routine implementation of optical technologies for image-guided surgery purposes.

## Data Availability

If not already present in the manuscript, all remaining datasets used during the current study are available from the corresponding author on reasonable request.

## References

[1] Gibbs SL (2012). Near infrared fluorescence for image-guided surgery. Quant Imaging Med Surg.

[2] Alander JT (2012). A review of indocyanine green fluorescent imaging in surgery. Int J Biomed Imaging.

[3] Rosenthal EL (2015). Safety and Tumor Specificity of Cetuximab-IRDye800 for Surgical Navigation in Head and Neck Cancer. Clin Cancer Res.

[4] de Valk KS (2019). A zwitterionic near-infrared fluorophore for real-time ureter identification during laparoscopic abdominopelvic surgery. Nat Commun.

[5] Folli S (1992). Immunophotodiagnosis of colon carcinomas in patients injected with fluoresceinated chimeric antibodies against carcinoembryonic antigen. Proc Natl Acad Sci U S A.

[6] Lee CM (2017). Sentinel node mapping using a fluorescent dye and visible light during laparoscopic gastrectomy for early gastric cancer: result of a prospective study from a single institute. Ann Surg.

[7] Sun JY (2014). In vivo optical imaging of folate receptor-β in head and neck squamous cell carcinoma. Laryngoscope.

[8] van Dam GM (2011). Intraoperative tumor-specific fluorescence imaging in ovarian cancer by folate receptor-α targeting: first in-human results. Nat Med.

[9] van den Berg NS (2017). Multispectral fluorescence imaging during robot-assisted laparoscopic sentinel node biopsy: a first step towards a fluorescence-based anatomic roadmap. Eur Urol.

[10] van Leeuwen FW, Hardwick JC, van Erkel AR (2015). Luminescence-based imaging approaches in the field of interventional molecular imaging. Radiology.

[11] Burggraaf J (2015). Detection of colorectal polyps in humans using an intravenously administered fluorescent peptide targeted against c-Met. Nat Med.

[12] Roberts DW (2018). Red-light excitation of protoporphyrin IX fluorescence for subsurface tumor detection. J Neurosurg.

[13] Yeung TM (2016). Identifying ureters in situ under fluorescence during laparoscopic and open colorectal surgery. Ann Surg.

[14] Gioux S, Choi HS, Frangioni JV (2010). Image-guided surgery using invisible near-infrared light: fundamentals of clinical translation. Mol Imaging.

[15] van Beurden F (2020). Multi-wavelength Fluorescence in image-guided surgery, clinical feasibility and future perspectives. Mol Imaging.

[16] van Willigen DM (2017). Multispectral fluorescence guided surgery; a feasibility study in a phantom using a clinical-grade laparoscopic camera system. Am J Nucl Med Mol Imaging.

[17] Acerbi F (2016). Feasibility of simultaneous sodium fluorescein and indocyanine green injection in neurosurgical procedures. Clin Neurol Neurosurg.

[18] Della Puppa A (2019). Combined fluorescence using 5-aminolevulinic acid and fluorescein sodium at glioblastoma border: intraoperative findings and histopathologic data about 3 newly diagnosed consecutive cases. World Neurosurg.

[19] Kaibori M (2016). Intraoperative detection of superficial liver tumors by fluorescence imaging using indocyanine green and 5-aminolevulinic acid. Anticancer Res.

[20] Schwake M (2015). Simultaneous fluorescein sodium and 5-ALA in fluorescence-guided glioma surgery. Acta Neurochir (Wien).

[21] Yano H (2017). Pathological analysis of the surgical margins of resected glioblastomas excised using photodynamic visualization with both 5-aminolevulinic acid and fluorescein sodium. J Neurooncol.

[22] Suero Molina E (2018). Dual-labeling with 5-aminolevulinic acid and fluorescein for fluorescence-guided resection of high-grade gliomas: technical note. J Neurosurg.

[23] Meershoek P (2018). Multispectral-fluorescence imaging as a tool to separate healthy from disease-related lymphatic anatomy during robot-assisted laparoscopy. J Nucl Med.

[24] Moore FA (2015) Full spectrum LED illuminator. Google Patents

[25] Berezin MY, Achilefu S (2010). Fluorescence lifetime measurements and biological imaging. Chem Rev.

[26] van der Wal S (2016). Synthesis and systematic evaluation of symmetric sulfonated centrally CC bonded cyanine near-infrared dyes for protein labelling. Dyes Pigment.

[27] Sjöback R, Nygren J, Kubista M (1995). Absorption and fluorescence properties of fluorescein. Spectrochim Acta Part A Mol Biomol Spectrosc.

[28] Benson RC, Kues HA (1978). Fluorescence properties of indocyanine green as related to angiography. Phys Med Biol.

[29] Chin PTK (2013). Optical imaging as an expansion of nuclear medicine: cerenkov-based luminescence vs. fluorescence-based luminescence. Eur J Nucl Med Mol Imaging.

[30] KleinJan GH (2016). Fluorescence guided surgery and tracer-dose, fact or fiction?. Eur J Nucl Med Mol Imaging.

[31] Laios A (2015). A prospective pilot study of detection of sentinel lymph nodes in gynaecological cancers using a novel near infrared fluorescence imaging system. BMC Res Notes.

[32] Lane B, Bohnstedt BN, Cohen-Gadol AA (2015). A prospective comparative study of microscope-integrated intraoperative fluorescein and indocyanine videoangiography for clip ligation of complex cerebral aneurysms. J Neurosurg.

[33] Shahid MW (2011). Exploring the optimal fluorescein dose in probe-based confocal laser endomicroscopy for colonic imaging. J Interv Gastroenterol.

[34] Manny TB, Patel M, Hemal AK (2014). Fluorescence-enhanced robotic radical prostatectomy using real-time lymphangiography and tissue marking with percutaneous injection of unconjugated indocyanine green: the initial clinical experience in 50 patients. Eur Urol.

[35] Schottelius M (2019). Synthesis and preclinical characterization of the PSMA-targeted hybrid tracer PSMA-I&F for nuclear and fluorescence imaging of prostate cancer. J Nucl Med.

